# High-*k* Solution-Processed Barium Titanate/Polysiloxane Nanocomposite for Low-Temperature Ferroelectric Thin-Film Transistors

**DOI:** 10.1021/acsomega.2c08142

**Published:** 2023-08-09

**Authors:** Aimi Syairah Safaruddin, Juan Paolo S. Bermundo, Chuanjun Wu, Mutsunori Uenuma, Atsuko Yamamoto, Mutsumi Kimura, Yukiharu Uraoka

**Affiliations:** † Division of Materials Science, Nara Institute of Science and Technology, Nara 630-0192, Japan; ‡ Display Solutions Patterning Materials, Merck Electronics Ltd., Shizuoka 437-1412, Japan; § Department of Electronics and Informatics, Ryukoku University, Seta 520-2194, Japan

## Abstract

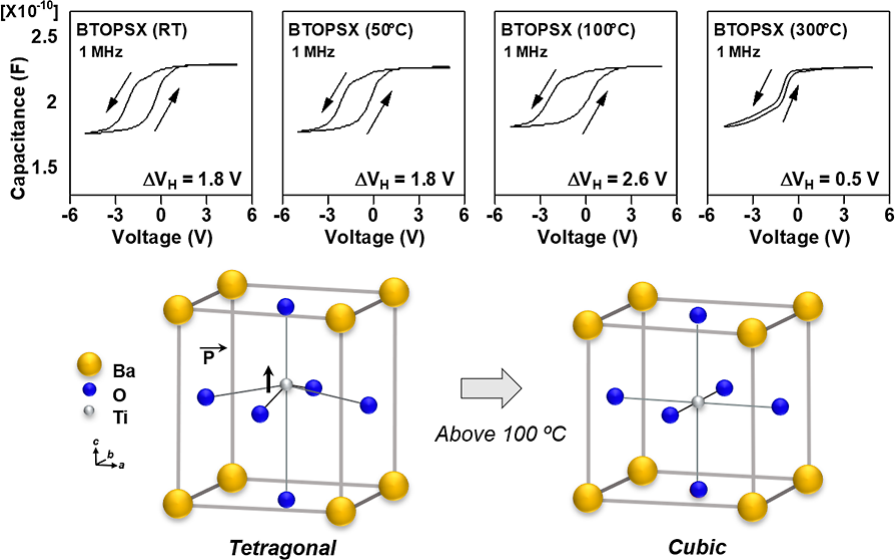

Ferroelectric nanoparticles have attracted much attention for numerous electronic applications owing to their nanoscale structure and size-dependent behavior. Barium titanate (BTO) nanoparticles with two different sizes (20 and 100 nm) were synthesized and mixed with a polysiloxane (PSX) polymer forming a nanocomposite solution for high-*k* nanodielectric films. Transition from the ferroelectric to paraelectric phase of BTO with different nanoparticle dimensions was evaluated through variable-temperature X-ray diffraction measurement accompanied by electrical analysis using capacitor structures. A symmetric single 200 peak was constantly detected at different measurement temperatures for the 20 nm BTO sample, marking a stable cubic crystal structure. 100 nm BTO on the other hand shows splitting of 200/002 peaks correlating to a tetragonal crystal form which further merged, thus forming a single 200 peak at higher temperatures. Smaller BTO dimension exhibits clockwise hysteresis in capacitance–voltage measurement and correlates to a cubic crystal structure which possesses paraelectric properties. Bigger BTO dimension in contrast, demonstrates counterclockwise hysteresis owing to their tetragonal crystal form. Through further Rietveld refinement analysis, we found that the tetragonality (*c*/*a*) of 100 nm BTO decreases at a higher temperature which narrows the hysteresis window. A wider hysteresis window was observed when utilizing 100 nm BTO compared to 20 nm BTO even at a lower loading ratio. The present findings imply different hysteresis mechanisms for BTO nanoparticles with varying dimensions which is crucial in understanding the role of how the BTO size tunes the crystal structures for integration in thin-film transistor devices.

## Introduction

Ferroelectric ceramics denote the group of dielectric materials which possesses spontaneous polarization property in which the dipole orientation of the material will align depending on the applied voltage. Several ceramic materials with ferroelectric properties have been developed which mostly consist of the perovskite family group of ABX_3_ such as barium titanate (BaTiO_3_), lead zirconate titanate (PZT), and lead titanate (PbTiO_3_) for non-volatile memories, piezoelectric sensors, and actuators, as well as data storage application.^1−4^ Perovskite-type ceramic exists in several crystal structures which tuned its properties against an external electric field.^5^ Among all, barium titanate (BaTiO_3_ or BTO) has been extensively studied due to its non-toxicity (Pb-free) with low dielectric loss, ferroelectric phase at room temperature, and high dielectric constant with strong scaling capability. BTO has been explored in various device application such as multilayer ceramic capacitors, photovoltaic cells, as well as utilization as gate dielectrics for thin-film transistors (TFTs). BTO is a ferroelectric oxide with Curie temperature (*T*_C_) of 120 °C which undergoes phase transition depending on the subjected temperature.^6^ BTO can exist in several crystal forms: tetragonal, cubic, hexagonal, orthorhombic, and rhombohedral. Among these phases, cubic phase possesses paraelectric property, while tetragonal, orthorhombic, and rhombohedral have ferroelectric property.^7^ Remarkably, with various crystal structures, BTO exhibits useful properties for different electronic applications. It has been known that the simple yet ideal cubic (*Pm* 3̅*m*) BTO can be slightly distorted depending on the fabrication temperature, size changes, and their chemical composition. The tetragonal (*P*4*mm*) phase was found to be stable at room temperature which provides important characteristics especially in the electronic industry as one of the promising candidates for ferroelectric and piezoelectric application. Interestingly, the ferroelectric property in BTO is naturally formed at a lower temperature without requiring complex experimental process or additional annealing treatment which open up the possibility of using this material for integrated applications in flexible and ubiquitous devices. The ferroelectric property of BTO material is triggered through off-center shift or displacement of Ti^4+^ ions from the centrosymmetric position which leads to the formation of an electrical dipole.^8^ The alignment in the electrical dipole creates spontaneous polarization which depends on the symmetry of the unit cell which correlated to the degree of the tetragonality and nanocrystal size. The ferroelectric tetragonal BTO have been widely utilized for ceramic capacitor devices due to their remnant polarization as well as hysteresis loop area.^9^

Our previous work has demonstrated that the incorporation of high-*k* BTO nanoparticles into a polymer to form a polymer nanocomposite is an effective gate insulator film for high performance amorphous oxide semiconductor (AOS) TFTs.^10^ AOS TFTs are competent components for scalable-area electronics due to their attractive features of high mobility, low fabrication temperature, solution-process compatibility, and flexibility.^11^ Numerous research has been performed to explore the potential of AOS TFT devices for various electronic purposes such as display, memory, and various sensors applications.^12^ In addition, great effort has been implemented to improve the device performance such as achieving low operating voltage for commercial applications with various approaches.^13−15^ One approach is by increasing the areal capacitance of the gate insulator layer which potentially lowers the operating voltage and increase the accumulated carriers. The method of utilizing high-*k* polymer nanocomposites results on high dielectric constant, inexpensive manufacturing costs, and large-area processing.^16^ From our prior finding, the contribution of high-*k* BTO as gate insulator successfully enhanced the electrical characteristics by improving the field effect mobility as well as subthreshold swing value.^17^ Additionally, dispersing the BTO nanoparticles in low-*k* polymer matrix helps in lowering the overall leakage current, improving surface morphology, with better film flexibility compared to when employing the nanoparticles solely as gate insulator layer. Motivated by our previous work, we investigated the possibility of mixing polymer solution with different nanoparticle sizes for TFT application. To this end, BTO nanoparticles has been utilized in different research studies and various electronic applications depending on their crystal structure. In this work, we first report the temperature and size dependence studies of BTO nanoparticles embedded in a hybrid polysiloxane (PSX) polymer as gate insulator layer which aims to retain the ferroelectric property for ferroelectric TFTs (Fe-TFTs) application. Fe-TFT is suitable in memory devices due to its smaller cell size with excellent device stability.^18^ The polarization state of the ferroelectric layer controls the accumulation or depletion state of oxide channel depending on the external voltage applied. By utilizing the BTOPSX nanocomposite, tunable properties could be achieved through various BTO-to-PSX ratios along with diverse nanoparticle sizes which manifest changes in physical and electrical properties. Additionally, the structure of polymer matrix was modified based on the incorporated alkyl/aryl groups which tuned the film characteristics such as hydrophobicity, film density, and crosslinking temperature. This work demonstrates the pronounced effect that different crystal sizes has on the direction of hysteresis loop from metal–insulator–semiconductor (MIS) capacitor. The ferroelectric property was successfully achieved upon shifting to larger particle dimension. The calculated *c*/*a* ratio decreased with smaller nanoparticle dimension which results in lower probabilities of polarization phenomenon. The mechanism of the ferroelectricity was further confirmed with various temperature conditions and described with variable temperature X-ray diffraction (VTXRD) analysis alongside with the behavior observed in MIS capacitor.

## Results and Discussion

### Precursors and Surface Morphology

**Figure 1:**
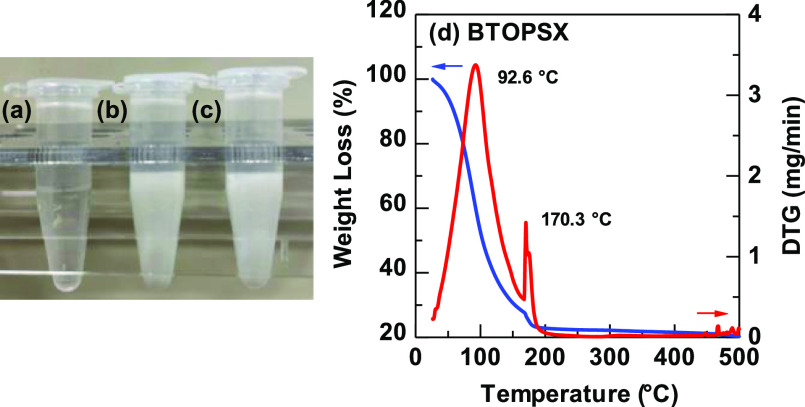
(a) PSX solution, (b) BTOPSX nanocomposite solution with BTO nanoparticles dispersed in PSX solution, (c) BTO solution, and (d) TG-DSC of BTOPSX solution.

**Figure 2:**
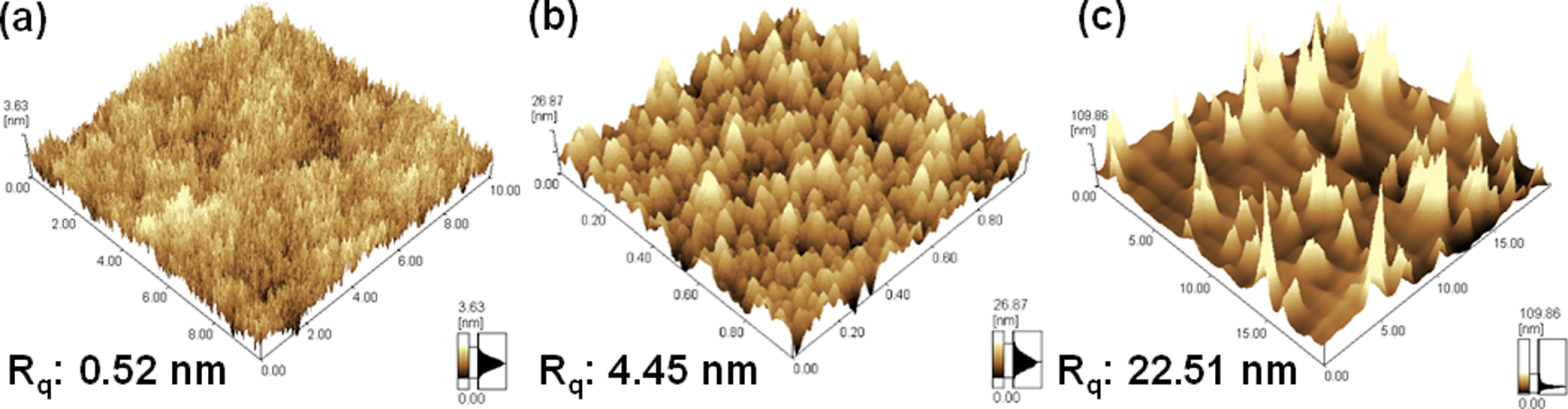
Three-dimensional (3D) topography images of (a) PSX, (b) BTOPSX20-3, and (c) BTOPSX100 nanocomposite films.

The separately prepared BTO (20 and 100 nm) nanoparticles and PSX solution were mixed and stirred until a homogeneous solution was obtained to form BTOPSX nanocomposite solution, as shown in Figure 1b. Thermogravimetry and differential scanning calorimetry (TG-DSC) analysis was carried out to determine the solvent evaporation temperature for the complete polymerization of the PSX polymer. Figure 1d shows the TG-DSC curves of BTOPSX solution showing the weight loss temperature of BTOPSX solvent at 92.6 °C and the film formation temperature at 170.3 °C. Figure 2 compares the three-dimensional surface topography of the PSX polymer and BTOPSX nanocomposite films. The displayed results revealed that the BTO nanoparticles with 20 nm size can be distributed well. However, 100 nm nanoparticle samples showed nonuniformity in the BTO distribution which affected the roughness of the film, as shown in Figure 2c. 100 nm BTOPSX sample suffers substandard film roughness of 22.51 nm due to the poor nanoparticle distribution as well as agglomeration. 20 nm nanoparticle BTOPSX samples on the other hand revealed excellent nanoparticle distribution with a smoother film roughness of 4.45 nm. Energy-dispersive X-ray spectroscopy (EDX) as well as top-view field emission scanning electron microscopy (FE-SEM) images presented in Figure S1 dictates the element presents in the BTOPSX100 film corresponded to different areas in the film. The area with BTO nanoparticles displayed barium and titanium detection, and the PSX region is presented by Si detection. The region consisting BTO nanoparticles was chosen to be electrically evaluated for capacitor behavior.

### Electrical Characteristics of BTOPSX Thin Film

**Figure 3:**
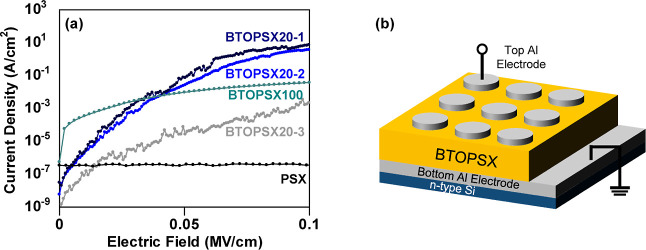
*J*–*E* characteristics of (a) MIM for PSX, 20 nm BTOPSX20 with different BTO ratios, BTOPSX100 for 100 nm dimension, and (b) schematic of the MIM structure.

The electrical characteristics of BTOPSX films with different nanoparticle sizes were assessed using metal–insulator–metal (MIM) structure, and the dielectric constant was measured following eq 1 utilizing the MIS structure, where *C* is the maximum capacitance value of the films, ε is the relative permittivity which is equal to *k* (dielectric constant) × ε_o_ (vacuum permittivity), *A* is the area of the measured electrode, and *d* is the thickness of the BTOPSX film (see Figure S2). 20 nm BTO nanoparticles were incorporated in PSX solution with BTO ratios of 70, 60, and 50%. Meanwhile, BTOPSX100 was prepared utilizing 100 nm size with lower loading ratio of 20%. The dielectric constant for pure PSX polymer was calculated using 100 μm diameter size electrodes, and the values are tabulated in Table 1. The BTO nanoparticles were successfully integrated into the PSX polymer as manifested by a higher dielectric constant of BTOPSX samples with 20 nm BTO nanoparticles at different loading ratios. The dielectric constant values are in the 5–11 range, larger than the dielectric constant of PSX polymer, which is *k* = 2.25, due to the contribution of BTO nanoparticles. Figure 3 shows the leakage current density against electric field (*J*–*E*) measurement from MIM of different BTOPSX samples. The *J*–*E* results show that the leakage current tends to increase at higher BTO loading ratio in 20 nm BTO. On the other hand, a 20% loading ratio of 100 nm BTO also has increased leakage current with value higher than the 20 nm BTO with 50% loading ratio. High leakage in the 100 nm BTOPSX film was attributed to the non-uniformity and agglomeration observed when having a big BTO dimension. Nanocomposite materials are frequently employed as the gate insulator layer in the presence of inorganic particles to increase the capacitance value and the organic polymer provides smooth surface roughness. Introducing higher BTO loading ratio for the 100 nm sample increases its dielectric constant to be comparable with 20 nm BTO. Nevertheless, higher composition of 100 nm BTO led to a worse film roughness which further deteriorated the leakage current. To be employed as a ferroelectric material for FeFET applications, leakage current of the gate insulator layer should be controlled as it could be one of the major causes that yield to a shorter retention time which further deteriorates the memory state. Therefore, 20% 100 nm BTO was embedded in the PSX polymer as the optimized loading ratio for 100 nm nanocomposite film.
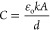
1

**Table 1:** Comparison of *k*-Value Calculated from MIM Devices with the Calculated Leakage Current Density Extracted from *I*–*V* Measurement

samples	Np size (nm)	thickness (nm)	BTO ratio (%)	roughness, Rq(nm)	*k*-value	leakage current density (A cm^–^^2^)
PSX		1000		0.92	2.25	4.54 × 10^–^^7^
BTOPSX20-3			50	4.45	5.11	3.55 × 10^–^^3^
BTOPSX20-2	20	100–200	60	4.70	7.59	4.05 × 10^0^
BTOPSX20-1			70	5.28	11.3	7.78 × 10^0^
BTOPSX100	100	300–400	20	22.51	5.56	3.77 × 10^–^^2^

**Figure 4:**
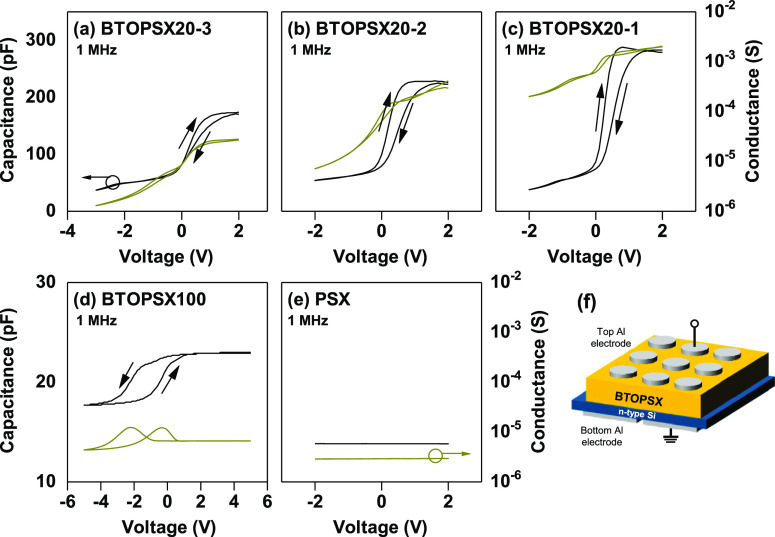
*C*–*V* characteristic comparing BTOPSX20 with different BTO ratios of (a) 50%, (b) 60%, and (c) 70%, BTOPSX100 with (d) 20%, (e) PSX only film, and MOS (f) capacitor structure used.

In addition to investigating the dielectric properties by varying the nanoparticles sizes, we also examined the device performance utilizing MIS capacitor structures which were prepared to analyze and compare the hysteresis characteristics of BTOPSX incorporated with different nanoparticle sizes. Capacitance–voltage (*C*–*V*) measurement was executed at 1 MHz and the hysteresis was estimated between forward and reverse sweeps. The voltage sweeps were fixed at ±2 V for all samples despite their difference in thickness due to different dielectric breakdown strengths. Figure 4 shows the *C*–*V* characteristics comparing the hysteresis of all samples measured in room temperature and dark environment. An apparent improvement in device hysteresis was observed when comparing PSX only device with BTOPSX nanocomposite devices. Research have shown the advantage of integrating BTO nanoparticles in polymer matrix which helped in improving the memory window by either acting as electron trapping center or enhancing the ferroelectric property which induces a better polarized state.^19,20^ In our finding, the roles of BTO nanoparticles were distinguished based on the hysteresis direction of MIS capacitors. Incorporation of 20 nm BTO with various BTO loading ratio was performed to elucidate the difference in hysteresis window. 20 nm BTO significantly enhanced the hysteresis window as compared to PSX devices with an improvement in clockwise hysteresis shift (Δ*V*_H_) of 0.5 V compared to PSX with 0 V. Clockwise hysteresis (CW) direction was primarily associated to charge trapping mechanism which was widely studied for non-volatile memory application.^21−23^ Within BTO nanoparticles, there are many defects such as oxygen vacancies and positively charged traps due to ionic bonding that has poor ability to remove the defects. The incorporation of 20 nm BTO loading successfully lead to the Δ*V*_H_ by Ti^4+^ acting as electron trapping sites thus contributing to a wider hysteresis window due to its lower ionization energy. Higher 20 nm BTO loading ratio improved the trapping characteristics manifested by a wider clockwise Δ*V*_H_ when comparing between 50 and 70% loading ratios. Nevertheless, even with the incorporation of higher 20 nm BTO loading, the Δ*V*_H_ is still insufficient for reliable memory application.

Interestingly, 100 nm BTO devices exhibited wider hysteresis with a Δ*V*_H_ of 1.4 V even at a lower loading ratio. Besides, it is worthwhile to note that the hysteresis direction of the BTO samples is opposite that of BTOPSX20 despite BTOPSX100 having the same base material. The hysteresis direction of 100 nm BTO samples revealed counterclockwise (CCW) direction which correlates to the polarization of the ferroelectric film via dipole alignment of BTO nanoparticles.^24,25^ Previous finding has verified that spontaneous polarization of BTO nanoparticles was derived from the asymmetric position of Ti atom in tetragonal crystal structure which possesses ferroelectric property.^26^ Our finding revealed that the device mechanism of BTO capacitors are greatly affected by the dimension of the BTO nanoparticles exhibited by the difference in hysteresis direction. The dominant mechanism for smaller BTO nanoparticles is correlated to charge trapping characteristics which improved with higher BTO loading. Meanwhile, hysteresis observed for the bigger BTO dimension was interrelated to spontaneous polarization results from the tetragonal crystal structure.

### Crystal Phase of Different BTO Dimensions

BTO commonly demonstrates ferroelectric properties in orthorhombic, tetragonal, and rhombohedral phases except for cubic crystal phase.^6^ Previous experimental and theoretical studies have shown that the phase transition of BTO is interrelated to the nanoparticle dimension and compositional variation which affect the ferroelectric behavior.^27−30^ Several characterizations were proven to successfully distinguish the crystal phase of the BTO nanoparticles such as micro-Raman spectrometer and X-ray diffraction (XRD) analysis. Therefore, to gain an insight into the crystal phase of BTO nanoparticles, variable-temperature X-ray diffraction (VTXRD) analysis was executed to examine the different crystal structures of the BTOPSX nanocomposite film. Different BTO powders were prepared and placed inside anton-paar DHS 1100 domed under a vacuum condition. The XRD spectra were recorded at different heating temperatures, namely, room temperature (RT), 50, 100, 110, 120, 130, 180, and 300 °C which were retained for an hour prior to the measurement. For the tetragonal phase, both 200 and 002 peaks can be clearly distinguished through splitting of both peaks and/or both peaks coexisting together resulting in peak broadening at 2θ of 45°.^31^ Meanwhile, the cubic BTO structure reflects a single symmetric peak at 45°.^32^

**Figure 5:**
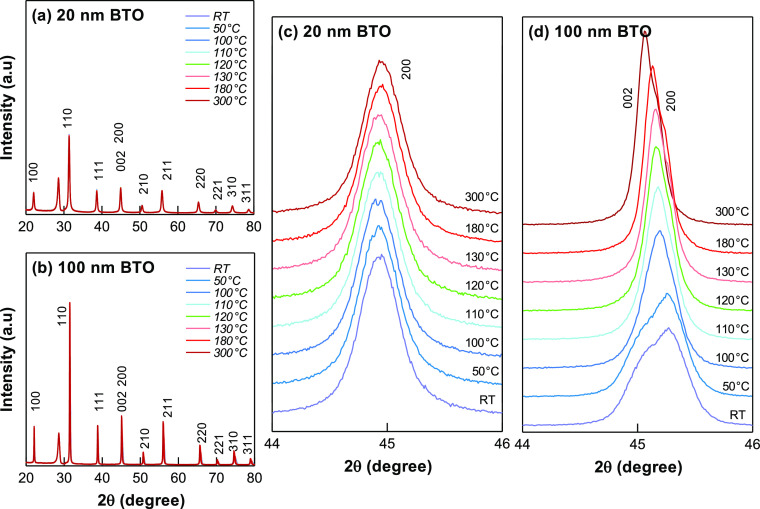
Variable-temperature XRD spectra of (a) BTOPSX1 and (b) BTOPSX2 with zoomed comparing the shift in the peaks in the 200/002 diffracted peaks for (c) BTOPSX1 and (d) BTOPSX2 at different temperatures.

Figure 5 shows that 20 nm prepared BTO powder revealed the cubic crystal phase with XRD spectra of a single 200 diffraction peak for RT measurement up to 300 °C. On the other hand, 100 nm BTO nanoparticles revealed different XRD spectra at different temperatures. The BTO sample was confirmed to be in tetragonal crystal form at low temperature with two sub-peaks existing together with a growing dominant 002 peak thus leading to broadening in 2θ = 45° as temperature is increased. Phase transition from tetragonal to the cubic crystal structure was visibly observed at 100–110 °C with the dominant peak shifting from 002 to 200 peak. The difference in full-width half maximum (FWHM) of 2θ angle of 45° was also compared, as shown in Table S2. A noticeable phase transition was confirmed from merging of 200 and 002 peaks forming one single peak above the Curie temperature (*T*_C_) which in this case is above 100 °C. The obtained data matched well with *P*4*mm* (tetragonal) and *Pm*3̅*m* (cubic) space groups depending on the subjected temperature.^33^

**Figure 6:**
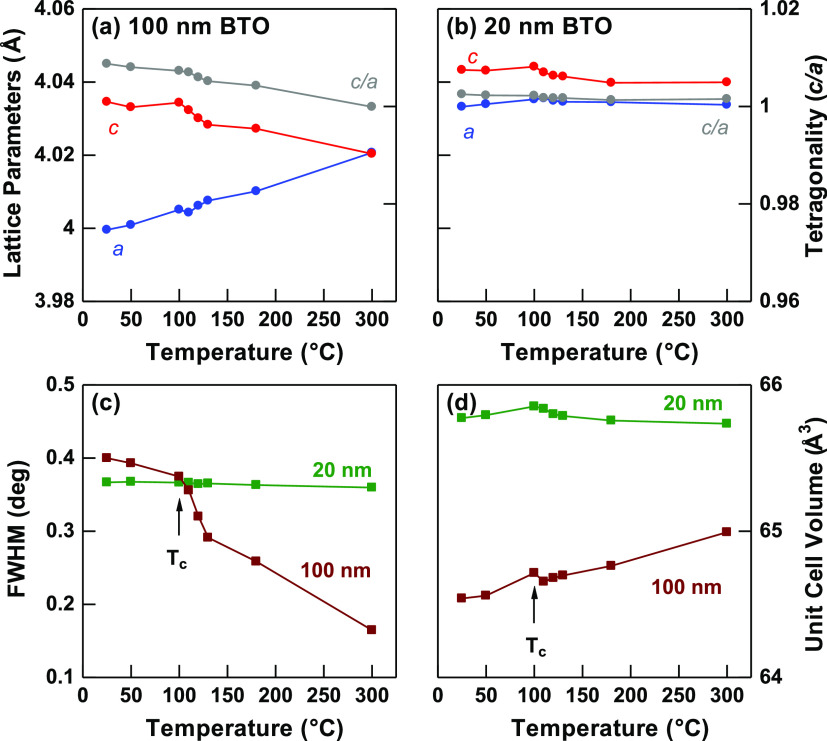
XRD parameters extracted for both samples with (a) FWHM at 2θ of 45°, with tetragonality difference at various temperatures for (b) 20 nm, and (c) 100 nm BTO powder, as well as (d) the unit cell volume.

Additionally, lattice parameter was calculated from the VTXRD diffraction data utilizing Rietveld refinement method. Rietveld reliability weighted profile denoted as *R*_wp_ was used to measure the square root of the difference between the measured intensity and calculated intensity which further act as indication of good fitting analysis. Figures S4 and S5 show the pattern-matching using tetragonal BTO phase (crystallography open database of 1507756) for all temperatures for 20 nm and 100 nm BTO samples. The degree of tetragonality (*c*/*a*) for both samples were plotted, as shown in Figure 6. 20 nm BTO marks almost constant *c*/*a* value close to 1.00 with almost negligible change in FWHM indicating cubic crystal phase verified through a symmetric single XRD peak. 100 nm BTO, on the other hand, exhibited gradual change in *a* and *c* parameters as temperature increases. The rate of change becomes the greatest with an abrupt fall in *c*/*a* value marking the phase transition temperature above 100 °C. This finding confirmed that larger BTO dimension has a different crystal structure which is expected to vary hysteresis direction observed from Figure 4d, unlike in smaller BTO dimension. Aside from lattice parameter, size dependence thermal expansion is further discussed considering the unit cell volume (UCV) of different BTO sizes. As the size reduces from 100 to 20 nm, the UCV increases as shown in Figure 6d which is consistent with previous report.^34^ It was also noticeable that the UCV for 20 nm has almost linear function with minimal change at different temperatures. The high and almost constant UCV for small BTO dimension correlates to the “lattice-softening” surface effect, generating an easy recovery of Ti atoms displacement from the center.^35^ 100 nm BTO on the other hand exhibited positive thermal expansion with an increase in UCV with elevated temperatures. This is due to weakening of long-range ferroelectric causing a shrink in *c* axis at higher temperature but cannot compensate the lengthening of the *a* axis, thus causing an increase in UCV in 100 nm BTO. The difference in *c*/*a* marks the degree of tetragonality for the 100 nm BTO sample.

### Ferroelectric BTOPSX Thin Film

**Figure 7:**
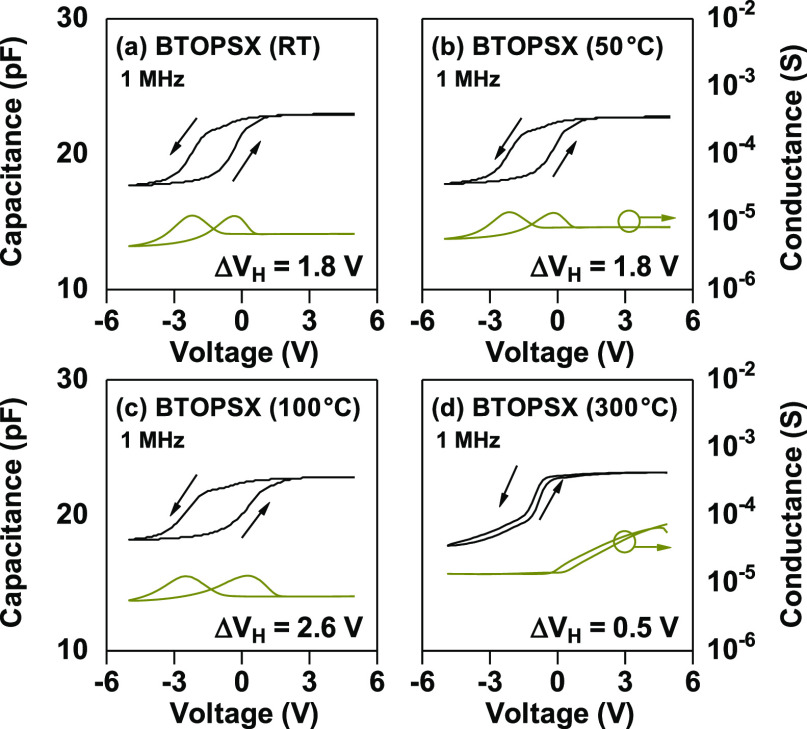
*C*–*V* characterization of 100 nm BTOPSX100 treated at four different temperatures: (a) RT, (b) 50 °C, (c) 100 °C, and (d) 300 °C treated MOS samples.

On the basis of preserving the ferroelectric property to benefit the wider memory window, 100 nm BTOPSX *C*–*V* measurement was executed again at 1 MHz sweeping voltage of ±5 V with BTOPSX100 cured at different temperatures to explore the effect of the crystal structure on the electrical characteristics. MIS capacitors were prepared with BTOPSX100 cured at RT, 50, 100, and 300 °C. Figure 7 shows different hysteresis behaviors of BTOPSX100 treated at different temperatures. Low-temperature-treated sample exhibited wider CCW hysteresis of 1.8, 1.8, 2.6, and 0.3 V for RT, 50, 100, and 300 °C, respectively. Sample treated at 300 °C revealed hysteresis narrowing behavior unlike the low-temperature treated samples. Our VTXRD demonstrated phase transition of 100 nm BTO above 100 °C which probably affects the hysteresis behavior. Below the *T*_C_, the CCW hysteresis window widened with an increase in curing temperature specifically above the solvent evaporation temperature (92.6 °C). This is due to the removal of precursor-related impurities which possibly act as trapping sites that lead to CW hysteresis nature which counteracts the hysteresis induced by ferroelectric BTO nanoparticles. Impurities originated from carbon in the solvent could act as electron capturing sites, thus shifting the threshold voltage positively during the voltage sweeps. With an increase in temperature above the solvent evaporation temperature and below *T*_C_, the hysteresis window widened from 1.8 to 2.6 V due to the removal of organic impurities while maintaining ferroelectricity from the tetragonal crystal nature. However, with a further increase in temperature above the *T*_C_, the hysteresis window almost vanishes due to the disappearance of ferroelectric switching properties as the BTO transforms into the cubic crystal phase. The wide hysteresis observed for low-temperature treated samples is owing to the tetragonal crystal structure which is well-known to possess ferroelectric property originated from the stretching or shrinking of Ti atom thus led to a spontaneous polarization. The direction of the *C*–*V* behavior also confirmed that the characteristics observed were due to polarization phenomenon as the hysteresis direction change from CW to CCW when the nanoparticle dimension was changed from 20 to 100 nm size.^36^

**Figure 8:**
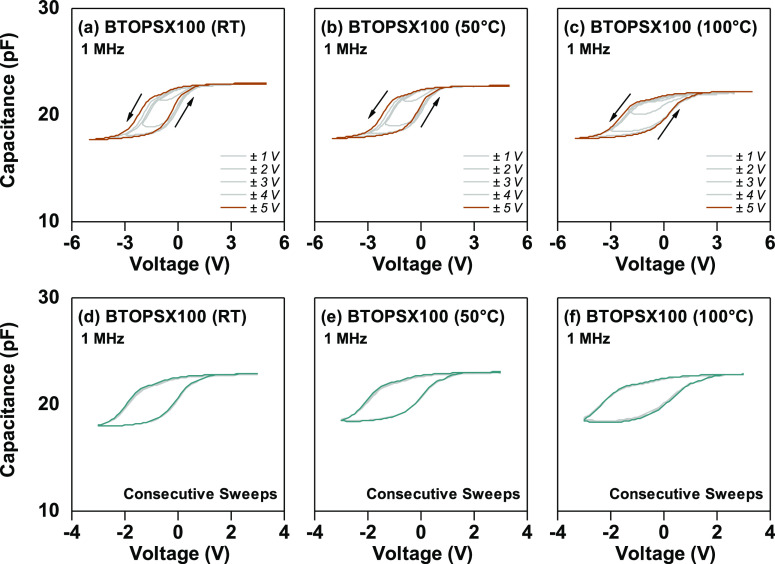
Hysteresis *C*–*V* loop characterized using the MIS structure of BTOPSX100 cured at low-temperature (a) RT, (b) 75 °C, and (c) 100 °C, with the stable consecutive sweeps of (d) RT, (e) 75 °C, and (f) 100 °C samples after five repeating cycles.

In order to investigate the effect of ferroelectric switching on BTOPSX nanocomposite films below their *T*_C_, the gate voltage of double *C*–*V* sweeps was continuously increased starting from (−1, 1 V) to (−5, 5 V) with an increment of 1 V as shown in Figure 8a–c. Δ*V*_H_ tends to increase with a larger sweeping voltage, as summarized in Table S4. An increase in sweeping voltage promotes better CCW memory window accompanied by the improvement in Δ*V*_H_. The Δ*V*_H_ of BTOPSX nanocomposite films increases from 0.1 to approximately 3 V due to the enhanced polarization switching factor under large external applied gate voltage accompanied by a better film formation at higher temperature. Aside from that, the ferroelectric behavior stability of an approximately 300 nm thick BTOPSX100 was examined through multiple sweeps *C*–*V* measurement, as shown in Figure 8d–f. Almost a negligible change in CCW direction with stable capacitance values is observed for all the measurements marking a fast switching between polarization and depolarization states of BTOPSX100 films under a short holding time of 50 ms. This result suggests that carrier accumulation and depletion are predominantly controlled by switching properties of nanocomposites films. Nevertheless, we believe, with higher number of cycles, the devices would be stable if the voltage applied is lower than the breakdown voltage for each sample. Beyond the voltage limit, the *C*–*V* loops might deteriorate with a high conductance value resulting from high leakage current.

**Figure 9:**
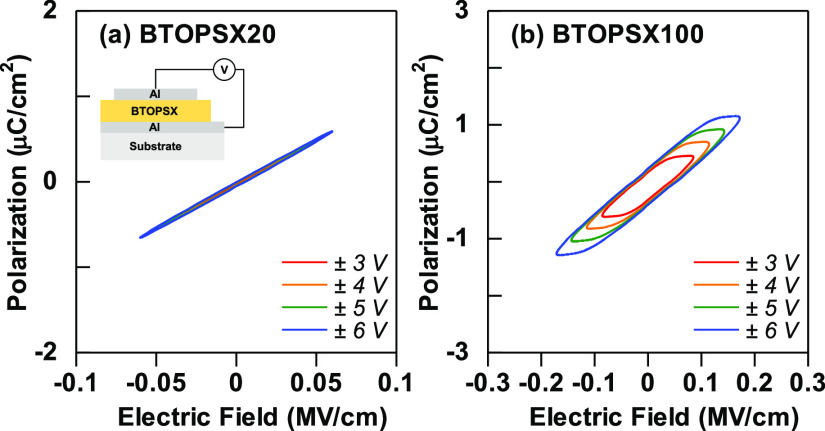
Polarization vs electric field hysteresis loop of (a) BTOPSX20, and (b) BTOPSX100. The inset presents the capacitor structure used for the measurement.

The ferroelectric property of tetragonal BTO nanoparticles was further confirmed through polarization against electrical field (*P*–*E*) loops measurement examined through the Sawyer–Tower circuit, as illustrated in Figure S3.^37^ The circuit comprises two capacitors connected in series, one is the sample which was deposited between two Al metal forming the MIM structure with a total thickness, *d* and electrode area, *A*. The other capacitor is the reference capacitor which was chosen to be significantly large to ensure that the voltage applied to the sample will be almost similar to the total voltage applied. The hysteresis loops were measured using the AC power supply at a set frequency of 1 kHz using sine waveform. The polarization as a function of electric field was then plotted based on the output data from the digital oscilloscope. Two different MIM samples were prepared to elucidate the difference in *P*–*E* loops comparing ferroelectric and paraelectric behaviors. BTOPSX100 and BTOPSX20 were chosen to elucidate the difference in the polarization against electric field of BTO samples with different crystal structures. Figure 9 presents the *P*–*E* loop comparing BTOPSX100 and BTOPSX20. Low-temperature-treated BTOPSX100 revealed semi-saturated hysteresis loop with remnant polarization (2*P*_r_) of 0.50 μC/cm^2^ and coercive field of 0.06 MV/cm proving the existence ferroelectric properties which originate from the tetragonal structure of BTO nanoparticles. Yet, the polarization state of BTOPSX100 is still considered low due to the high loading of the paraelectric PSX polymer which makes up 80% of the nanocomposite composition. BTOPSX20 on the other hand exhibited a linear capacitor behavior due to the cubic crystal structure which possesses paraelectric properties. *P*–*E* loop data further proved that the origin of the different in hysteresis directions from the *C*–*V* characteristics is dependent on the crystal structure of BTO nanoparticles. Wide hysteresis for low-temperature treated BTOPSX100 nanocomposite film was confirmed owing to the tetragonal structure of BTO nanoparticles arising from the shrinking of *c*-axis of the BTO structure. The off-centered position of Ti atoms lead to spontaneous polarization manifested by the hysteresis in the *P*–*E* loop as in Figure 9b.

### BTOPSX Thin-Film as Dielectric for Ferroelectric TFT Fabrication

**Figure 10:**
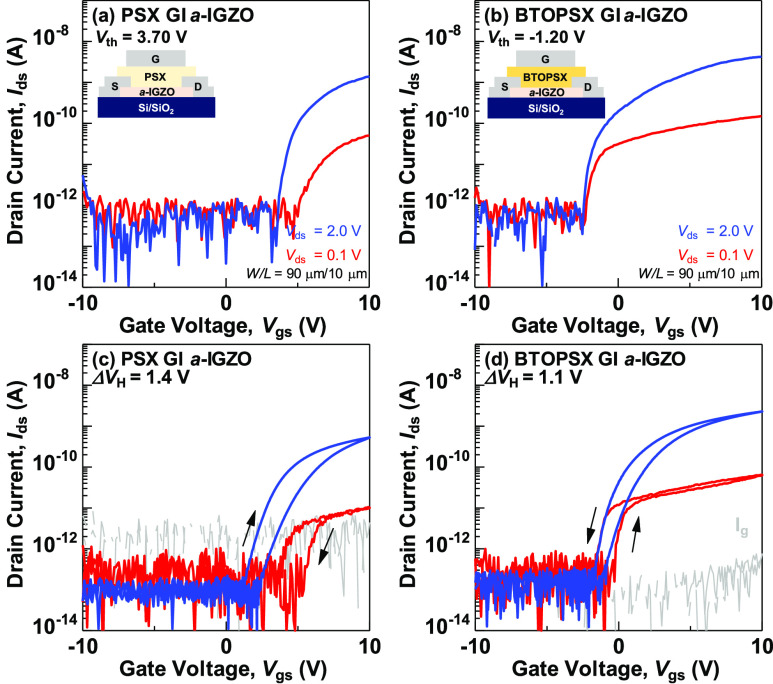
Transfer characteristics of a-IGZO TFTs with different gate insulator layers comparing (a) PSX (*k* = 2.25), and (b) BTOPSX100 (*k* = 5.66) with the inset of top-gate TFT structures employing both insulator films. Transfer characteristics in the forward and reverse sweeps of *V*_gs_ from −10 to +10 V of (c) PSX and (d) BTOPSX100 *a*-IGZO TFTs.

The standard bottom gate top contact AOS TFTs were fabricated by employing amorphous InGaZnO (*a*-IGZO) as the active channel layer. Then, BTOPSX100 and PSX materials were spin coated as the gate dielectric layer and cured for 1 h. The whole dielectric deposition layer is operated with a maximum temperature of 100 °C. Top gate electrode was subsequently deposited through electron beam heating. Transfer characteristics comparing *a*-IGZO TFT performance with BTOPSX nanocomposite and PSX gate insulators are presented in Figure 10. Both gate insulators illustrated low drain current due to lower annealing temperature applied to the sample. The TFT with BTOPSX, however, exhibited slightly higher drain current compared to PSX insulator with low-voltage function confirmed by the shift in *V*_th_ to a lower gate voltage below 2 V. Aside from that, different hysteresis direction was observed comparing both gate insulator layers. CW direction associated to the charge trapping characteristics near gate insulator and channel interfaces was observed in the PSX gate insulator sample. Meanwhile, TFTs with BTOPSX100 as the gate insulator layer exhibits CCW hysteresis with a hysteresis window of 1.1 V. The application of forward sweep (from low to high voltage) leads to an accumulation of electrons due to the polarization of the BTOPSX layer, resulting in an increase in drain current by 2 orders of magnitude. During reverse sweep (from high to low voltage), reduction in drain current was observed suggesting a channel depletion condition. This CCW directionality reported in this study is consistent with ferroelectric TFT theory.^38^ Hence, the CCW hysteresis suggests the existence of ferroelectricity attributed from the polarization switching of the BTOPSX gate insulator.

Aside from that, additional measurement with various sweeping voltage was conducted, and the hysteresis observed is summarized in Table S5. As the gate voltage increases, the CCW memory window of the devices also increases proving the polarization phenomenon of the BTOPSX100 layer. The hysteresis improved from 0.3 to 5.4 V at *V*_ds_ of 0.1 V for sweeping *V*_gs_ from ±1 to ±7 V. This suggests that the employed BTOPSX100 material is suitable as a candidate for ferroelectric applications targeted for low-operational voltage devices. Further improvement on the quality of the insulator layers could be explored such as by utilizing smaller tetragonal BTO sizes to improve the nanoparticles dispersion as well as maintaining thin gate insulator layer for faster polarization at lower applied voltage. Other than that, the nanoparticle modification or functionalization such as capping or adding dispersant can be used to lower the leakage current. This improvement could be employed to further enhance the device characteristics for future ferroelectric applications.

## Experimental Section

### Materials

The BTOPSX nanocomposite was prepared by solution mixing method. The PSX solution with alkyl compositional ratio of 70% silica and 30% methyl were mixed with BTO powder prepared by Toda Kogyo Corp. forming the nanocomposites blend formulations. Two different nanoparticles sizes were studied (20, and 100 nm) with different volume ratios as in Table 1. The solution was stirred using a Vortex mixer with rpm of 1500 for 1 h to obtain a homogeneous solution prior to the deposition process. The nanocomposite films were then deposited via a spin coating technique with a syringe filter to inhibit nanoparticle agglomeration. Various filters have been explored in depositing a continuous thin film with minimum surface roughness. The film thickness ranged from 100 to 400 nm depending on nanoparticles size, as measured by cross-sectional SEM observation in Figure S2.

### TFT Fabrication

The Si/SiO_2_ substrates (0.001∼0.007 Ω cm) were pre-cleaned using sulfuric acid and hydrogen peroxide mixture solution for 10 min each. Subsequently, the 70 nm thick *a*-IGZO channel was deposited at room temperature through radio-frequency (RF) magnetron sputtering using a target composition of In:Ga:Zn:O = 2:2:1:7 (atomic ratio) at 100 W RF power with a mixture of Ar + O_2_ gas. The channel pattern was then formed through photolithography and wet etching process using 0.02 M HCl solution. 80 nm thick titanium (Ti) and 20 nm thick gold (Au) metal were deposited as source and drain electrodes via the electron beam heating method. The *a*-IGZO TFTs were annealed at 300 °C for 2 h in an atmospheric environment (N_2_/O_2_ 4:1). PSX and BTOPSX100 solution were filtered through syringe filters prior to the deposition via spin coating technique as the gate insulator layer. Both films were subjected to a maximum of 100 °C temperature for 1 h as the post-baking process. Then, 100 nm thick aluminum (Al) metal was deposited as top contact gate electrode. All aforementioned processes were performed in a controlled clean room environment.

### Metal–Insulator–Semiconductor

The n-type Si (0.3∼0.8 Ω cm) substrates were pre-cleaned using sulfuric acid and hydrogen peroxide mixture solution for 10 min each. The 3 nm thick native SiO_2_ layer was then etched through wet etching process with BHF solution. 100 nm thick bottom gate Al electrode was deposited and patterned on the back side of n-type Si wafer, followed by annealing in a N_2_ environment for 30 min at 400 °C. Subsequently, PSX and BTOPSX solution was filtered through syringe filters prior to the deposition of BTOPSX film via spin coating technique. The samples were then treated at different maximum curing temperatures. 100 nm thick Al electrodes were deposited and patterned using metal mask through electron beam evaporation as the top electrodes. The stacked Al/BTOPSX/Si/Al structure was used to evaluate the hysteresis characteristics.

### Metal–Insulator–Metal

Filtered BTOPSX solution was deposited on Al metal coated on highly doped Si/SiO_2_ substrates (0.001∼0.007 Ω cm). Subsequently, the 100 nm thick Al top contacts were then deposited on the BTOPSX and PSX films.

### Sample Characterizations

TG-DSC (Hitachi DSC7000X/STA7200) was performed to identify the decomposition temperature of BTOPSX solution. In addition, VTXRD measurement was carried out at various temperature using an X-ray structure analyzer (Rigaku SmartLab9kW/IP/HY/N) equipped with a Cu Kα X-ray source. Additionally, ultrahigh resolution field emission scanning electron microscopy (FE-SEM, Hitachi SU9000) and atomic force microscopy (AFM) were utilized to observe the film morphology and topography as well as nanoparticle size estimation. Ferroelectric polarization of the films was measured through the Sawyer–Tower circuit utilizing a mixed signal oscilloscope (MSO/DPO2000B) and a waveform generator (Keysight 33511B). The dielectric constant and hysteresis of nanocomposite films was estimated from capacitance against voltage (*C*–*V*) measurement using precision LCR meter Agilent E4980A. The electrical characteristics of the bottom gate top contact *a*-IGZO TFTs as well as leakage current were measured in the dark condition with a semiconductor device parameter analyzer (Agilent 4156C) at an ambient environment.

## Conclusions

In summary, two different BTO nanoparticle sizes were explored for high-*k* dielectric films for ferroelectric TFT applications. BTO with 20 nm nanoparticle dimension shows a stable cubic crystal phase with the CW hysteresis direction owing to the charge trapping as the dominant hysteresis mechanism. 100 nm BTO in contrast demonstrated CCW hysteresis due to its tetragonal crystal form which exhibited ferroelectric properties. Thermal expansion for individual BTO nanoparticles demonstrates an easy recovery of Ti shifting for 20 nm BTO samples unlike the 100 nm BTO nanoparticle. The 100 nm BTO exhibits spontaneous polarization which derived from the asymmetric position of the Ti atom in the tetragonal structure. The shifting or off-centering position of Ti atoms along the *c*-axis resulted in dipole orientation within the nanoparticles which induced the spontaneous polarization. *P*–*E* loop measurement was conducted in validating the ferroelectricity within the BTOPSX100 film. These results indicate that multiple behaviors of BTO nanoparticles at different dimensions in which bigger BTO nanoparticles are prone to show ferroelectric properties owing to their tetragonal crystal form and slowly changing to paraelectric properties above their *T*_C_. Meanwhile, smaller BTO dimension will have stable paraelectric properties due to cubic crystal forms even below *T*_C_. These findings show important structural insights of BTO nanoparticles which is beneficial for their integration in Fe-TFTs.

## Supporting Information Available

FE-SEM images with EDX elemental mapping of the surface of the nanocomposite BTOPSX film; additional cross-section SEM images included to estimate the thickness of PSX, BTOPSX20-1, BTOPSX20-2, and BTOPSX20-3, as well as BTOPSX100; Sawyer–Tower circuit employed to reveal the ferroelectric property of BTOPSX material; Rietveld refinement fitting of 20 nm BTO powder with the black line indicating experimental data, red line as fitted data, and blue line denotes residual spectra of difference between the fitted and experimental line; and Rietveld refinement fitting of 100 nm BTO powder with the black line indicating experimental data, red line as fitted data, and blue line denotes residual spectra of difference between the fitted and experimental line (PDF)
