# Supplementary material for: High-k Solution-Processed Barium Titanate/Polysiloxane Nanocomposite for Low-Temperature Ferroelectric Thin-Film Transistors
Source: ACS Omega. 2023 Aug 9;8(33):29939–48. doi: 10.1021/acsomega.2c08142 (PMC10448671; doi:10.1021/acsomega.2c08142)
Supplement: Supplementary file 1 — ao2c08142_si_001.pdf [file ao2c08142_si_001.pdf]

Supplementary Information

High-*k* Solution-Processed Barium Titanate/Polysiloxane Nanocomposite for Low-Temperature Ferroelectric Thin-Film Transistors

Aimi Syairah Safaruddin[1], Juan Paolo S. Bermundo[1,*], Chuanjun Wu[1], Mutsunori Uenuma[1], Atsuko Yamamoto[2], Mutsumi Kimura[3], and Yukiharu Uraoka[1]

[1]Division of Materials Science, Nara Institute of Science and Technology, Nara Japan

[2]Display Solutions Patterning Materials, Merck Electronics Ltd., Shizuoka Japan

[3]Department of Electronics and Informatics, Ryukoku University, Seta Japan

Corresponding Author

*E-mail: b-soria@ms.naist.jp

Division of Materials Science, Nara Institute of Science and Technology, Nara Japan

Table S1. Comparison of FWHM of 20 nm and 100 nm BTO from RT to 300 °C.

	Measurement Temperature (°C)							
	RT	50	100	110	120	130	180	300
FWHM 20 nm	0.3668	0.3676	0.3665	0.3662	0.3644	0.3653	0.3632	0.3597
FWHM 100 nm	0.4000	0.3930	0.3747	0.3558	0.3201	0.2912	0.2586	0.1647

Table S2. Parameters extracted for 20 nm BTO nanoparticles.

20 nm	Measurement Temperature (°C)							
	RT	50	100	110	120	130	180	300
FWHM	0.3668	0.3676	0.3665	0.3662	0.3644	0.3653	0.3632	0.3597
a	4.0333	4.0340	4.0353	4.0355	4.0350	4.0346	4.0345	4.0338
c	4.0433	4.0431	4.0442	4.0426	4.0417	4.0415	4.0398	4.0399
c/a	1.0025	1.0023	1.0022	1.0000	1.0017	1.0017	1.0013	1.0015
UCV (Å)	65.77	65.79	65.85	65.84	65.80	65.79	65.76	65.74
R_{wp} (%)	5.8964	5.8409	5.7149	5.6217	5.6212	5.5941	5.4662	5.4723

Table S3. Parameters extracted for 100 nm BTO nanoparticles.

100 nm	Measurement Temperature (°C)							
	RT	50	100	110	120	130	180	300
FWHM	0.4000	0.3930	0.3747	0.3558	0.3201	0.2912	0.2586	0.1647
a	3.9996	4.0009	4.0051	4.0042	4.0061	4.0076	4.0102	4.0207
c	4.0346	4.0331	4.0344	4.0323	4.0301	4.0283	4.0272	4.0204
c/a	1.0088	1.0081	1.0073	1.0070	1.0060	1.0052	1.0043	0.9999
UCV (Å)	64.54	64.56	64.71	64.66	64.68	64.70	64.76	64.99
R_{wp} (%)	6.4346	6.3700	6.3676	6.4585	6.6554	6.6523	6.8503	6.9811

Table S4. Summary of ΔV_H of low-temperature treated BTOPSX nanocomposite films at different sweeping voltages.

Sweeping Voltage	Hysteresis window, ΔV_H (V)				
	± 1 V	± 2 V	± 3 V	± 4 V	± 5 V
BTOPSX100 RT	0.1	1.4	1.6	1.7	1.8
BTOPSX100 50 °C	0.1	1.5	1.7	1.8	1.8
BTOPSX100 100 °C	0.6	1.3	2.2	2.4	2.6

Table S5. Summary of ΔV_H of BTOPSX GI for *a*-IGZO TFTs with various sweeping voltages.

Drain Voltage (V)	Hysteresis window, ΔV_H (V)						
	± 1 V	± 2 V	± 3 V	± 4 V	± 5 V	± 6 V	± 7 V
0.1	0.3	0.7	1.0	1.0	2.1	3.8	5.4
2	0.1	0.1	0.1	0.2	1.2	3.0	4.2

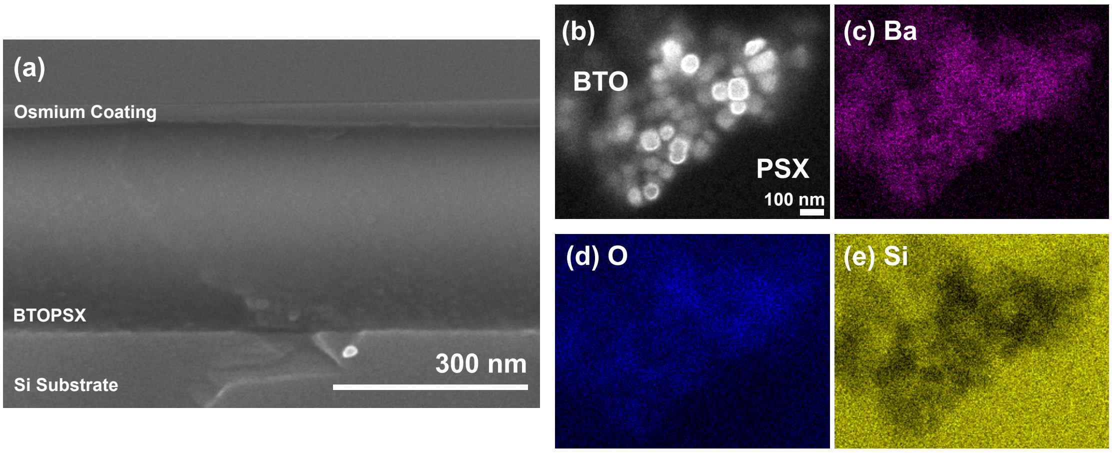

Figure S1. (a) Cross-sectional and top views (b) SEM images with EDX elemental mapping comparing (c) barium (Ba), (d) oxygen (O), (e) and silicon (Si) elements of BTOPSX100 nanocomposites film.

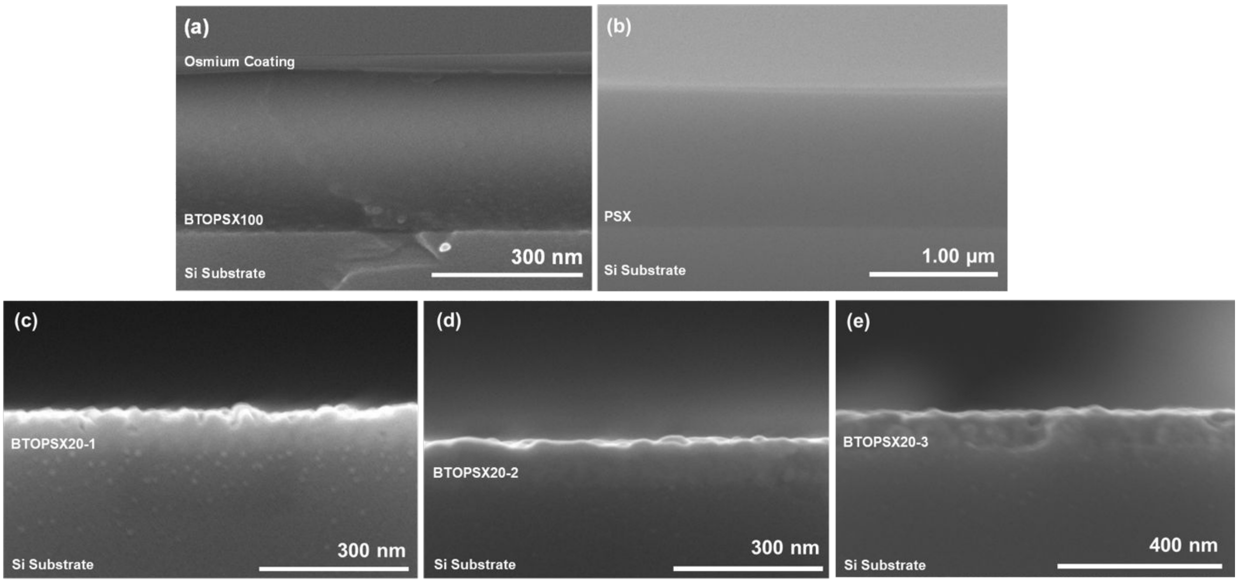

Figure S2. Cross-sectional SEM images comparing the thickness of (a) BTOPSX100, (b) PSX, (c) BTOPSX20-1, (d) BTOPSX20-2, and (e) BTOPSX20-3.

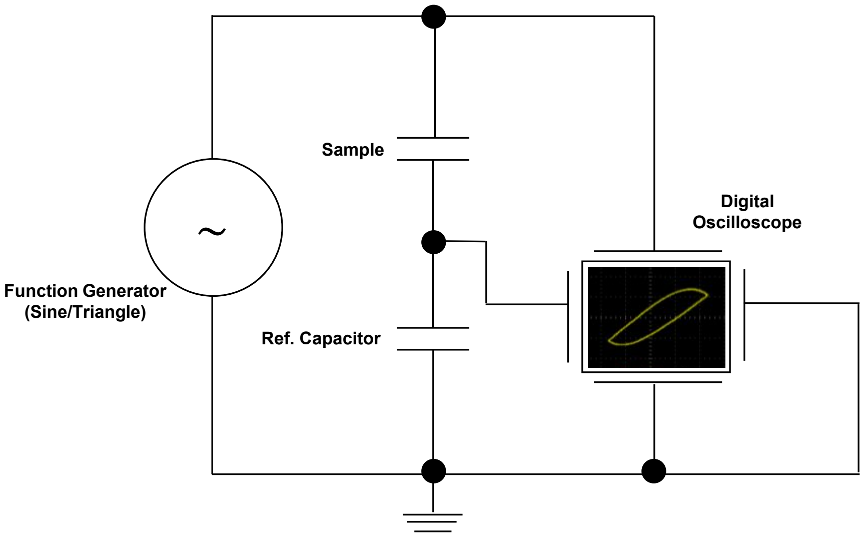

Figure S3. Illustration of Sawyer-Tower circuit design used to extract P-E loops measurement.

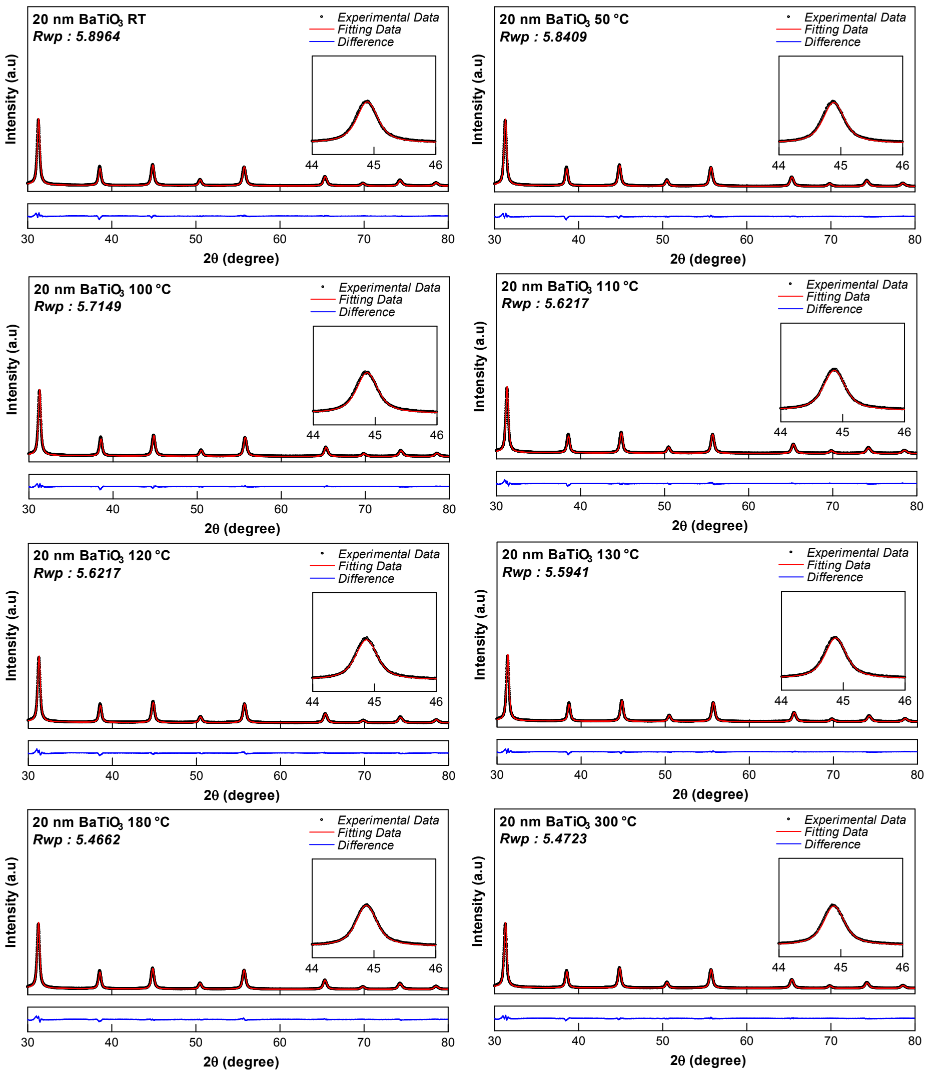

Figure S4. Rietveld refinement fitting of 20 nm BTO powder with black line indicating experimental data, red line as fitted data, and blue line denotes as residual spectra of difference between fitted and experimental line.

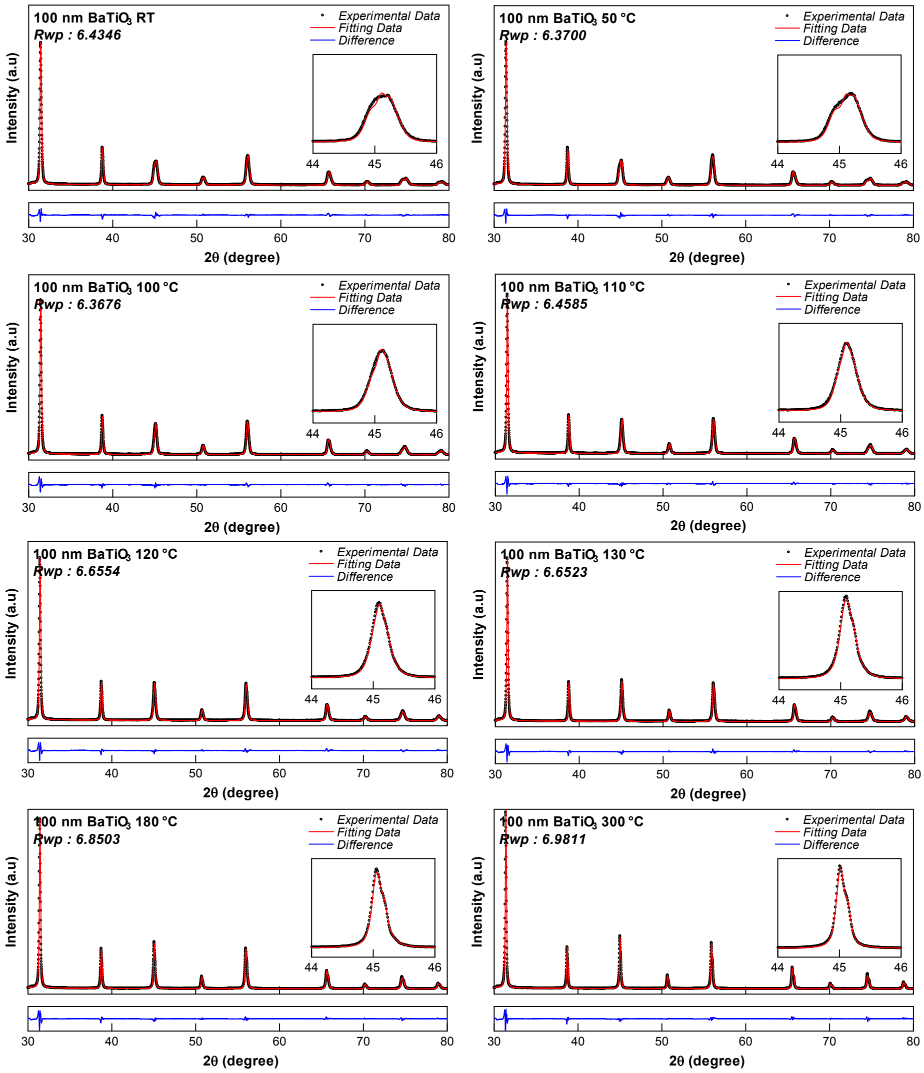

Figure S5. Rietveld refinement fitting of 100 nm BTO powder with black line indicating experimental data, red line as fitted data, and blue line denotes as residual spectra of difference between fitted and experimental line.
